# Supplementary material for: An Agrobacterium strain auxotrophic for methionine is useful for switchgrass transformation
Source: Transgenic Res. 2022 Oct 14;31(6):661–76. doi: 10.1007/s11248-022-00328-4 (PMC9747830; doi:10.1007/s11248-022-00328-4)
Supplement: Supplementary file 1 — Supplementary file1 (PDF 319 KB) [file 11248_2022_328_MOESM1_ESM.pdf]

Supplementary Information

Transgenic Research

An *Agrobacterium* strain auxotrophic for methionine is useful for switchgrass transformation

Mónica Prías-Blanco[1,2], Timothy M. Chappell[1], Emily F. Freed[3], Eudald Illa-Berenguer[4], Carrie A. Eckert[3,5], and Wayne A. Parrott[1,4,6*]

[1]Institute of Plant Breeding, Genetics & Genomics, University of Georgia, Athens, GA, USA

[2]International Center for Tropical Agriculture (CIAT), Km 17 Recta Cali-Palmira, P.O. Box 6713, Cali, Colombia

[3]Renewable and Sustainable Energy Institute (RASEI), University of Colorado Boulder, Boulder, CO, USA

[4]Center for Applied Genetic Technologies, University of Georgia, Athens, GA, USA

[5]Biosciences Division, Oak Ridge National Laboratory, Oak Ridge, TN, USA

[6]Department of Crop & Soil Sciences, University of Georgia, Athens, GA, USA

*Correspondence email: wparrott@uga.edu

Supplementary Table 1: Oligonucleotides used for assembling suicide plasmids and to identify and screen for auxotrophic *Agrobacterium* strains and pCAMBIA-1305.2 plasmid.

Name	Primer sequence (5´to 3´)	Purpose
EHA105_metA_seqF	GGGATTATGAAACGCCTCAGTGGTG	Confirmation of EHA105-derived auxotrophic mutant for methionine
EHA105_metA_seqR	TGACTTTCGCCGTCAAACCGTC	Confirmation of EHA105-derived auxotrophic mutant for methionine
LBA4404_metA_seqF1	CCTTATCGCACCTCAGGCAA	Confirmation of LBA4404-derived auxotrophic mutant for methionine
LBA4404_metA_seqR1	GGTGATAACCTTGGCCGTCA	Confirmation of LBA4404-derived auxotrophic mutant for methionine
HygR-For	CGTGCTTTCAGCTTCGATGTAG	Confirmation of T-DNA insertion
HygR-Rev	AAGATGTTGGCGACCTCGTATT	Confirmation of T-DNA insertion
EHA105_metA_FlankA_F	GGGGACAAGTTTGTACAAAAAAGCAGGCTTCTCTACCTGGAGGTTGGCACCGT	Amplify homology arm DNA from EHA105 genomic DNA.
EHA105_metA_FlankA_R	CTCGAGCCCGGGACTAGTGATCGGCATGTCGAACTCTCTTTTCC	Amplify homology arm DNA from EHA105 genomic DNA.
EHA105_metA_FlankB_F	ACTAGTCCCGGGCTCGAGGCGGCGTAAACTGCGGGATTG	Amplify homology arm DNA from EHA105 genomic DNA.
EHA105_metA_FlankB_R	GGGGACCACTTTGTACAAGAAAGCTGGGTCCTGCGTTTCAAGGCAGAAACCG	Amplify homology arm DNA from EHA105 genomic DNA.
LBA4404_metA_FlankA_F	GGGGACAAGTTTGTACAAAAAAGCAGGCTTCTCACGCCGAAGGTCAGCTTGAT	Amplify homology arm DNA from LBA4404 genomic DNA.
LBA4404_metA_FlankA_R	CTCGAGCCCGGGACTAGTGATCGGCATGTCGAACTCTCTTTTC	Amplify homology arm DNA from LBA4404 genomic DNA.
LBA4404_metA_FlankB_F	ACTAGTCCCGGGCTCGAGGCGTAAATTGCGGGATTGCGAATTG	Amplify homology arm DNA from LBA4404 genomic DNA.
LBA4404_metA_FlankB_R	GGGGACCACTTTGTACAAGAAAGCTGGGTCGGCCATATCTGCGTTTCCAGACA	Amplify homology arm DNA from LBA4404 genomic DNA.
GusPlus_ddPCR_F2	TGAAAATCTACACGACCCCG	Transgene detection for digital droplet PCR
GusPlus_ddPCR_R2	GCTTTGCCTTGAAAGTCCAC	Transgene detection for digital droplet PCR
GusPlus_ddPCR_Probe	[HEX]TCTCGGTTGTGACCGACTTCAATG[BHQ1]	Transgene detection for digital droplet PCR
PNPdPCR For	CGACTACACCAGGAGGCT	Reference gene for digital droplet PCR in switchgrass
PNPdPCR Rev	GTACCACACGGCGTTGAG	Reference gene for digital droplet PCR in switchgrass
PNPdPCR probe	[6FAM]CTCAACTTCACAGACTTCATCGTC[BHQ1]	Reference gene for digital droplet PCR in switchgrass

Abbreviations: HEX: Hexachloro-fluorescein; 6FAM: 6-Carboxyfluorescein; and BHQ1: Black Hole Quencher-1

Supplementary Table 2: Regions containing the gene necessary for methionine biosynthesis in EHA105 and LBA4404.

Strain	Deleted gene	Gene product	Locus tag	GenBank	Deficiency
EHA105	metA	Homoserine O-succinyltransferase	Atu2718	NC_003062 (2703611…2704537)	Methionine
LBA4404	metA	Homoserine O-succinyltransferase	Ach5_RS13190	NZ_CP011246 (2696107…2697033)	Methionine

Supplementary Table 3. Media used in tissue culture and transformation of Performer 7 switchgrass and TP309 rice.

Explant source	Name	Purpose	Macronutrients/ Micronutrients/ Vitamins	Hormones/others	Carbon source	Gelling agent	Reference
Transient transformation	mNB	Callus induction and maintenance)	N6/B5/B5	2,4-D (2 mg L^{-1})<br>L-Proline (500 mg L^{-1})<br>L-Glutamine (500 mg L^{-1})<br>Casein (300 mg L^{-1})	Sucrose (3% w/v)	Gelzan™ (0.25% w/v)	Chen et al. (1998)
Stable Transformation (switchgrass)	MSD5B0.15	Callus induction	MS/MS/MS	2,4-D (5 mg L^{-1})<br>BAP (0.15 mg L^{-1})	Sucrose (3% w/v)	Gelzan™ (0.25% w/v)	Xi et al. (2009)
	MSD5B1	Callus maintenance	MS/MS/MS	2,4-D (5 mg L^{-1})<br>BAP (1 mg L^{-1})	Maltose (3% w/v)	Gelzan™ (0.25% w/v)	Somleva et al. (2002)
	RMS-B1	Shoot regeneration	MS/MS/B5	BAP (1 mg L^{-1})	Sucrose (3% w/v)	Phytagel™ (0.25% w/v)	Alexandrova et al. (1996)
	½ MS-B5	Rooting	½MS/½MS/½B5		Sucrose (1.5% w/v)	Gelzan™ (0.25% w/v)	King et al. (2014)

Stable transformation (rice)	mNB	Callus induction and maintenance	N6/B5/B5	2,4-D (2 mg L^{-1})<br>L-Proline (500 mg L^{-1})<br>L-Glutamine (500 mg L^{-1})<br>Casein (300 mg L^{-1})	Sucrose (3% w/v)	Gelzan™ (0.25% w/v)	Chen et al. (1998)
	RGH6	Shoot regeneration	N6/N6/N6	BAP (3 mg L^{-1})<br>NAA (0.5 mg L^{-1})<br>L-Proline (500 mg L^{-1})<br>L-Glutamine (500 mg L^{-1})<br>Casein (300 mg L^{-1})	Sucrose (3% w/v)	Phytagel™ (0.25% w/v)	Broothaerts et al. (2005)
	½ MS-B5	Rooting	½MS/½MS/½B5		Sucrose (1.5% w/v)	Gelzan™ (0.25% w/v)	King et al. (2014)

Abbreviations: MS: Murashige & Skoog's medium (Murashige and Skoog 1962), B5: Gamborg's B5 medium (Gamborg et al. 1968), N6: Chu's N6 medium (Chu et al. 1975), mNB: modified NB (Chen et al. 1998), BAP: 6-benzylaminopurine, 2,4-D: 2,4-dichlorophenoxyacetic acid, NAA: 1-naphthaleneacetic acid.

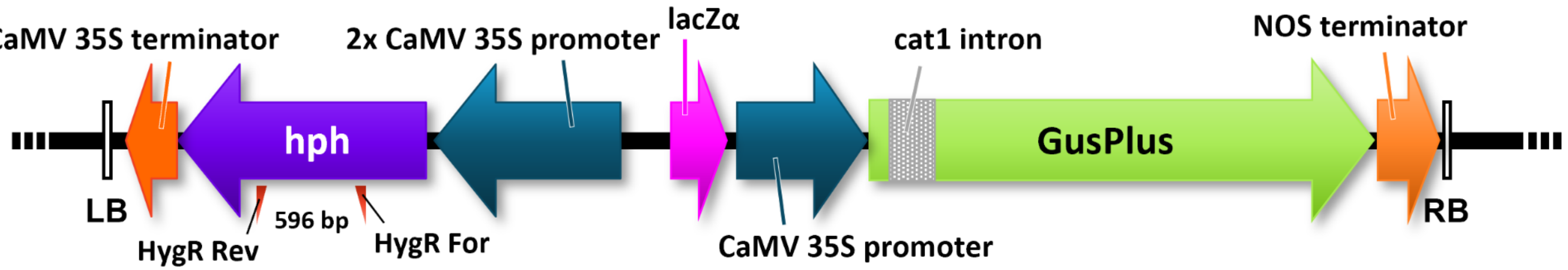

Supplementary Fig. 1 Schematic representation of T-DNA region of binary vector pCAMBIA-1305.2. Red triangles indicate the relative position of the PCR primers used to confirm T-DNA insertion. The expected PCR product size is indicated in space between the two primers.

References

Alexandrova KS, Denchev PD, Conger BV (1996) *In vitro* development of inflorescences from switchgrass nodal segments. Crop Sci. 36:175-178. https://doi.org/10.2135/cropsci1996.0011183X003600010031x

Broothaerts W, Mitchell HJ, Weir B, Kaines S, Smith LMA, Yang W, Mayer JE, Roa-Rodríguez C, Jefferson RA (2005) Gene transfer to plants by diverse species of bacteria. Nature 433:629-633. https://doi.org/10.1038/nature03309

Chen L, Zhang S, Beachy RN, Fauquet CM (1998) A protocol for consistent, large-scale production of fertile transgenic rice plants. Plant Cell Rep. 18:25-31. https://doi.org/10.1007/s002990050526

Chu C-C, Wang C-C, Sun C-S, Hsu C, Yin K-C, Bi F-Y (1975) Establishment of an efficient medium for anther culture of rice through comparative experiments on the nitrogen sources. Scientia Sinica 18:659-668.

Gamborg OL, Miller RA, Ojima K (1968) Nutrient requirements of suspension cultures of soybean root cells. Exp. Cell Res. 50:151-158. https://doi.org/10.1016/0014-4827(68)90403-5

King ZR, Bray AL, LaFayette PR, Parrott WA (2014) Biolistic transformation of elite genotypes of switchgrass (*Panicum virgatum* L.). Plant Cell Rep. 33:313-322. https://doi.org/10.1007/s00299-013-1531-1

Murashige T, Skoog F (1962) A revised medium for rapid growth and bio assays with tobacco tissue cultures. Physiol. Plant. 15:473-497. https://doi.org/10.1111/j.1399-3054.1962.tb08052.x

Somleva MN, Tomaszewski Z, Conger BV (2002) *Agrobacterium*-mediated genetic transformation of switchgrass. Crop Sci. 42:2080-2087. https://doi.org/10.2135/cropsci2002.2080

Xi Y, Fu C, Ge Y, Nandakumar R, Hisano H, Bouton J, Wang Z-Y (2009) *Agrobacterium*-mediated transformation of switchgrass and inheritance of the transgenes. Bioenerg. Res. 2:275-283. https://doi.org/10.1007/s12155-009-9049-7
